# Disturbed Experience of Time in Depression—Evidence from Content Analysis

**DOI:** 10.3389/fnhum.2018.00066

**Published:** 2018-02-20

**Authors:** David H. V. Vogel, Katharina Krämer, Theresa Schoofs, Christian Kupke, Kai Vogeley

**Affiliations:** ^1^Department of Psychiatry, University of Cologne, Cologne, Germany; ^2^Department of Psychiatry, Society for Philosophy and Sciences of the Psyche, Charité - Universitätsmedizin Berlin, Humboldt - Universität zu Berlin, Berlin, Germany; ^3^Institute for Neuroscience and Medicine, Cognitive Neuroscience (INM-3), Research Centre Juelich, Juelich, Germany

**Keywords:** depression, time experience, temporality, phenomenological psychopathology, content analysis

## Abstract

Disturbances in the experience of time have been a commonly reported feature of depressive disorders since the beginning of modern psychiatry and psychological research. However, qualitative research approaches to investigate the phenomenon are rarely used. We employed content analysis to investigate disturbances of time experience in Major Depressive Disorder. Our analysis from 25 participants showed that individuals with Major Depressive Disorder subjectively seem to have lost the ability to influence or change the present, resulting in an impersonal and blocked future. The present is rendered meaningless, the past unchangeably negative, and the passage of time turned into a dragging, inexorable, and viscous continuance. The overall,—possibly intersubjective—concept of time experience, remains largely intact, causing or adding to depressive mood and suffering. We elaborate on how these findings reflect previous theories on the experience of time in depression. This study might encourage future inquiries into both the phenomenal and neuroscientific foundation of time experience under psychopathological conditions.

## Introduction

Temporal dysfunctions are a commonly reported feature of Major Depressive Disorder (MDD). A variety of experimental approaches has been employed in order to assess and investigate these disturbances, with varying conclusions. For instance, research exploring human time perception has yielded inconsistent results concerning its potential disturbance in MDD (Thönes and Oberfeld, 2015). One of the more robust results from this line of research is the observation of a subjective decrease in the experienced velocity of the flow of time in depressive disorders. This decrease is possibly the most prominent and most often reported disturbance of time experience in MDD in the literature. It was either obtained from patients' qualitative descriptions (Lewis, 1932; Hartocollis, 1975; Wyrick and Wyrick, 1977; Kuhs, 1991; Mundt et al., 1998; Bschor et al., 2004; Stanghellini et al., 2016, 2017) or by making additional use of questionnaires or subjective quantitative measures (Lehmann, 1967; Bech, 1975; Kitamura and Kumar, 1982; Richter and Benzenhoefer, 1985; Blewet, 1992).

In this context phenomenological psychopathology offers a unique opportunity. Structural psychopathology investigating MDD appositely has identified disturbances in time experience as the basis underlying the disorder and linking its symptoms, ranging from impairments in neurocognitive and psychomotor functions, over changes in affect and mood to depressive delusions (Stanghellini et al., 2017). The emerging syndrome has adequately been referred to as a disturbance of *lived time* (Fuchs, 2001, 2013, 2014; Broome, 2005; Kupke, 2005; Wyllie, 2005; Gallagher, 2012; Moskalewicz, 2015; Bloc et al., 2016; Stanghellini et al., 2016), the slowing down of the subjective experience of time has been conceptualized to be a part of this syndrome (Wyllie, 2005; Fuchs, 2013, 2014; Stanghellini et al., 2017). The investigation of such an underlying principle or mechanism is of special significance, as current insights increasingly emphasize the importance of recognizing depressive symptoms apart from affect and mood for both diagnosis, therapy, and prognosis (Gonda et al., 2015). Despite the wealth of theoretical accounts on time experience, there has been only limited empirical research on the construct of *lived time* (Stanghellini et al., 2016, 2017). To the best of our knowledge, we present the first prospective empirical study employing qualitative content analysis as an empirical tool for phenomenological psychopathology to investigate disturbances of time experience in MDD. We will compare our results to those from a recent study on healthy individuals using the same method (Vogel et al., in revision).

## Materials and methods

### Participants

Participants were recruited from patients admitted for in-patient treatment to the Department of Psychiatry at the University Hospital Cologne. Patients were considered for inclusion into this study if the clinical diagnosis of a Severe Depressive Episode (ICD-10, F32.2) (World Health Organization, 1992) had been established after admission. Patients were only considered for inclusion, if they had neither record of comorbid neurological or comorbid psychiatric disease nor was there any neurological or psychiatric comorbidity detectable in the clinical diagnostic procedures. Comorbidities included organic brain disease, mental retardation (IQ < 70), manic or hypomanic episodes, bipolar disorder, psychosis or schizophrenia, personality disorders, and addiction. Patients eligible for inclusion were clinically rescreened approximately 2 weeks after admission by the principal investigators qualified for making a clinical diagnosis (DHVV, KV) for meeting both diagnostic criteria for a Severe Depressive Episode (ICD-10, F32.2) (World Health Organization, 1992) and diagnostic criteria for non-chronic MDD as defined by the DSM 5 (American Psychiatric Association, 2013). Patients were deemed eligible regardless of the number of prior depressive episodes, however patients diagnosed with chronic or persistent depressive disorder, or dysthymia were not considered for inclusion. Patients meeting these inclusion criteria were provided with the study material (see below). Patient screening and subsequent study inclusion were conducted over the course from January 2015 until August 2017. None of the patients included were taking any neuropsychiatric or any otherwise psychoactive or illegal drug not explicitly prescribed for anti-depressive treatment over the period of investigation. All participants received pharmacological anti-depressive treatment according to the S3 guidelines by the German Association for Psychiatry, Psychotherapy and Psychosomatics (Härter et al., 2017) (see Table 1). Two participants received electro-convulsive-therapy. Participants were included in a post-acute state, only after acute treatment interventions (e.g., treatment with benzodiazepines) had been successfully terminated. All participants underwent additional cognitive-behavioral therapy in group settings over the period of investigation.

**Table 1:** Prescribed daily medication and weekly Electro Convulsive Therapy (ECT).

D1	Mirtazapine 30 mg, Olanzapine 7.5 mg, Venlafaxine ret. 150 mg
D2	Mirtazapine 30 mg, Quetiapine 25 mg
D3	Venlafaxine ret. 225 mg
D4	Venlafaxine ret. 225 mg
D5	Bupropion 300 mg, Mirtazapine 15 mg
D6	Quetiapine ret. 300 mg, Agomelatine 50 mg, Tranylcypromine 50 mg, Lithium ret. 675 mg, Pregabalin 300 mg
D7	Lithium ret. 900 mg, Sertraline 150 mg
D8	Mirtazapine 30 mg
D9	Milnacipran 50 mg, Quetiapine 150 mg
D10	Mirtazapine 30 mg, Agomelatine 25 mg
D11	Escitalopram 10 mg
D12	Citalopram 20 mg, Quetiapine ret. 100 mg
D13	ECT, Tranylcypromine 40 mg
D14	Duloxetine 90 mg
D15	Duloxetine 90 mg
D16	Escitalopram 20 mg, Mirtazapine 30 mg
D17	Venlafaxine ret. 225 mg, Bupropion 150 mg
D18	Mirtazapine 30 mg, Venlafaxine ret. 150 mg, Pregabaline 200 mg
D19	Venlafaxine ret. 225 mg, Quetiapine 25 mg
D20	Venlafaxine ret. 225 mg
D21	Sertraline 200 mg, Trimipramine 75 mg
D22	Mirtazapine 30 mg
D23	Escitalopram 5 mg, Quetiapine ret. 300 mg, Lithium ret. 675 mg
D24	ECT, Venlafaxine ret. 225 mg, Quetiapine 50 mg
D25	Mirtazapine 45 mg, Aripiprazole 5 mg

We administered the Beck-Depression-Inventory (BDI-II) (Hautzinger et al., 1995) and a verbal IQ measure (WST) (Schmidt and Metzler, 1992). We used BDI-Scores to approximate depressive symptom severity and used WST-Scores to guarantee verbal speech comprehension, proper production of written material, and sufficient intellectual capability to cope with the complex subject of time experience. Demographics and results from BDI-II and WST are presented in Table 2.

**Table 2:** Demographics (Standard Deviation in brackets).

Gender	m = 15; w = 10
Mean age	47 (±14)
Mean years of education	16 (±3.5)
Mean BDI	19 (±11)
Mean verbal IQ	101 (±8)

### The time questionnaire

**Figure 1:**
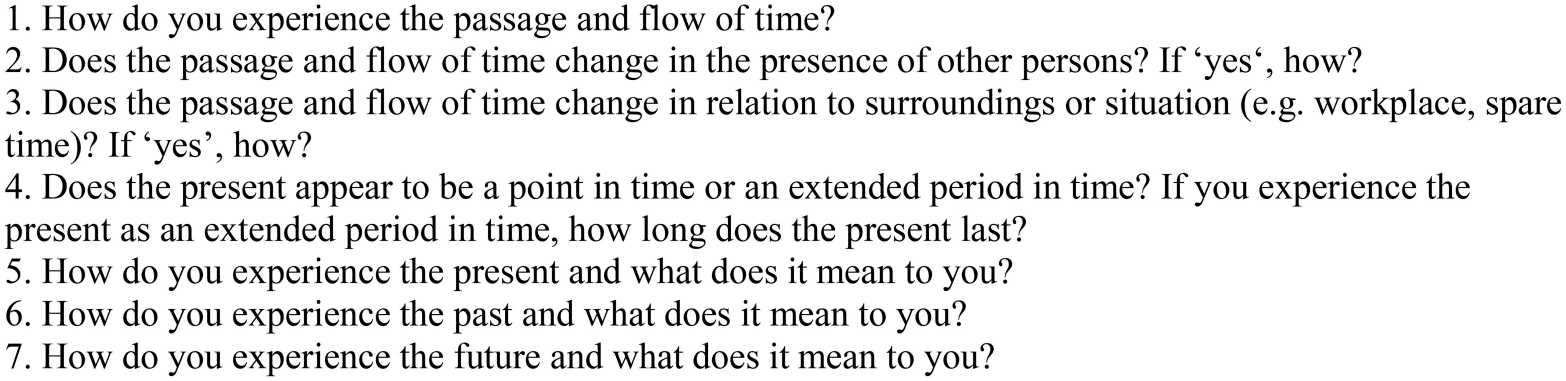
The Time Questionnaire.

The Time Questionnaire (TQ) (Figure 1) was specifically designed to address as many aspects of the experience of time as possible making use of open questions, and it has been successfully tested in a previous study on the experience of time in healthy participants (Vogel et al., in revision).

Questions (Q) 1–3 address the flow of time, with Q1 being as broad and open as possible and Q2–3 inquiring further into the dependency on interpersonal and situational context. Q4–7 are designed to address experience, concepts, and subjective meaning of the past, present, and future. Additional space is provided for participants to address anything they found relevant concerning the experience of time, but could in their opinion not be assigned to a specific question. Participants were instructed to write as much or as little as they found sufficient. There were no time limits for completion.

All returning documents were analyzed using *summarizing content analysis* (acc. to Mayring, 2014) (SCA). Superfluous examples and redundancies were deleted from the material. The remaining text was paraphrased into common language. The smallest possible part of the material to be categorized and coded was defined as a single statement within the material, ranging from a single word to multiple sentences. The largest possible part of the material that could form a coded category was defined as everything addressing one question. From the paraphrased material individual statements were identified. For each participant descriptive categories were generated for each single paraphrased statement. After all paraphrases had been assigned a category, the material was re-screened, and categories of similar or identical content were merged. This was repeated until a coherent categorical system had been established.

To evaluate the final categorical system's reliability, it was presented to two independent inter-coders (K.K., T.S.). Inter-coders were provided with an explication, a coding rule, and an anchor example and the code for each of the categories, together with the uncoded paraphrased material (Table 3). The inter-coders assigned each paraphrased statement to one category by employing the explication and coding rules. Inter-coder agreement was calculated by comparing codes from the coded material from the initial analysis, both inter-coders using Krippendorff's alpha (Krippendorff, 2004; Hayes and Krippendorff, 2007).

**Table 3:** The Categorical System.

K01	When active, time passes quickly.	“While at work, time passes quickly.”
K02	During pleasant situations/activities time passes quickly.	“The better I feel, the faster time passes”
K03	During unpleasant situations/activities time passes slowly.	“In an unpleasant atmosphere time passes slowly.”
K04	In the presence of others time passes slowly.	“In the presence of others time seems expanded”
K05	In the presence of others time passes quickly.	“Time passes more quickly when I am with others”
K06	In the presence of others the passage of time normalizes/feels more pleasant/is experienced more strongly.	“In the presence of others my sense of time normalizes.”
K07	Neither situations nor the presence of others influences the passage of time.	“While depressed, situations are not relevant for the passage of time.”
K08	The passage of time is meaningless/cannot be felt/sensed.	“The passage of time is meaningless.”
K09	The passage of time is unstoppable/endless/extended/a standstill.	“I get the feeling that I have to [act] against time.”; “Time is a merciless passage.”; “Time has stopped”
K10	Time passes quickly.	“Time passes very quickly.”
K11	Time passes slowly.	“Time drags.”
K12	The present is this day.	“The present is the current day.”
K13	The present is now.	“The present is the moment I am living in.”
K14	The present is associated with negative feelings.	“The present is evil and full of sorrow.”
K15	The present is the current activity.	“The present is while I say: ‘I am doing this or that’.”
K16	The present is not extended in time.	“The present seems like a point in time.”
K17	The present is extended in time.	“The present to me is an extended period in time.”
K18	The present cannot be influenced.	“The present is a burden because I am unable to act.”
K19	The present has to be utilized.	“The present is very important. What I enjoy I can take into the future.”
K20	The present is meaningless.	“Right now, the present is of lesser importance.”
K21	The past is felt with feeling of guilt. The past has a negative influence on the present.	“I live in the present. I constantly feel guilty for mistakes I have made in the past.”
K22	The past is over and unchangeable.	“The past to me is final, unchangeable.”
K23	The past has to be accepted.	“I try to forget the bad things from my past.”
K24	The past shapes an individual.	“I can use the past to learn.”
K25	The future is associated with fear, sorrow, and hopelessness.	“Anxiety concerning the future is common.”
K26	The future is blocked.	“Thinking about the future is difficult. It seems disconnected from the present moment.”
K27	The future is uncertain.	“The future is the great unknown.”
K28	The future may be influenced.	“The future is everything I have influence on right now.”
K29	The future is experienced with hope.	“The future is time of health, and I hope I will live to see it.”
K30	Rest.	“Little time produces stress.”

## Results

**Figure 2:**
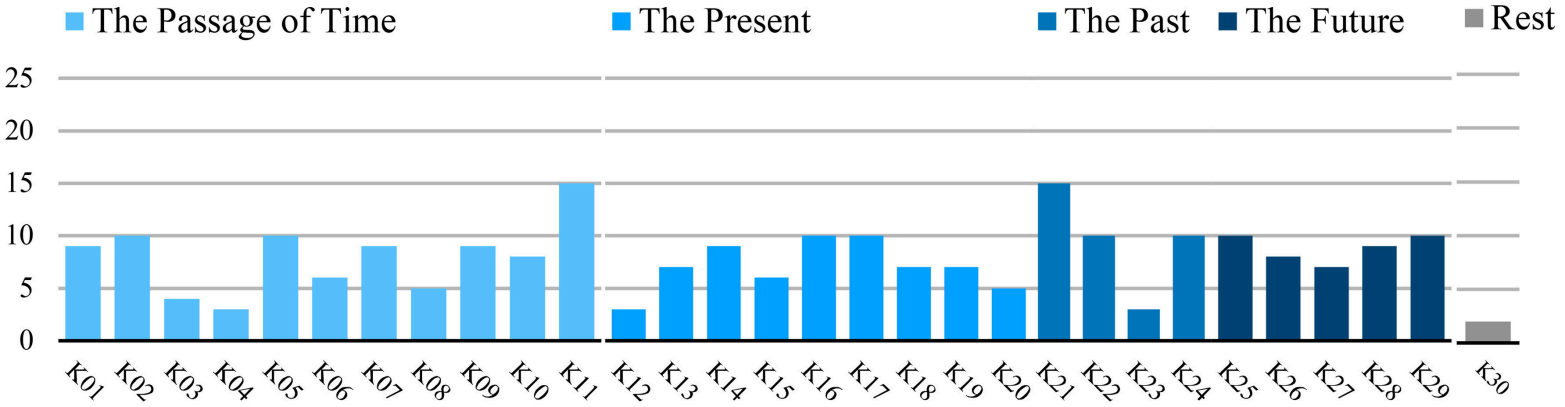
Category Count.

We identified 235 statements in total. We identified and explicated 30 categories which could be assigned to five different groups roughly corresponding to the initial formulation of questions. The first group consisted of eleven categories and addressed the passage of time (87 statements, app. 37% of all statements, categories K01-11). The second group consisted of nine categories concerning the present (64 statements, app. 27% of all statements, categories K12-20). The third group consisted of four categories and addressed the past (38 statements, app. 16% of all statements, categories K21-24). The fourth group addressing the future consisted of six categories (44 statements, app. 19% of all statements, categories K24-29). The last group was a single category labeled *Rest* (2 statements, app. 1% of all statements, category K30). This group coded for all statements which were not addressing the experience of time or were too unspecific or too divergent from the explanations of the other categories. The categorical system for the analyzed material is depicted in Table 3. The number of counts per category is depicted in Figure 2. Calculation of Krippendorff's alpha yielded a strong inter-coder agreement of α = 0.93.

### Category group “passage of time”

The majority of categories related to the Passage of Time concerned experiences of a changed velocity of passing time (categories 01, 02, 03, 04, 05, 10, 11; in total: *n* = 58 statements, app. 24% of all statements). Five categories related to changes in situational or social contexts (categories 01, 02, 03, 04, 05; in total: *n* = 36 statements, app. 15% of all statements). Most categories specified either an acceleration or an increase in the velocity of the passage of time during activities (category 01, *n* = 9 statements, 36% of participants), during engagement in pleasant situations (category 02, *n* = 10 statements, 40% of participants), or during engagement in interaction with others (category 05, *n* = 10 statements, 40% of participants). Fewer participants reported a deceleration or a decrease in the experienced velocity of the passage of time, namely during unpleasant situations (category 03, *n* = 4 statements, 16% of participants), or during the presence of others (category 04, *n* = 3 statements, 12% of participants). Two categories referred to general changes in the velocity of time irrespective of whether this experience was related to an activity (categories 10, 11; *n* = 18 statements, app. 9% of all statements), associated either with acceleration (category 10, *n* = 7 statements, 28% of participants) or a deceleration (category 11, *n* = 15 statements, 60% of participants). In the acceleration group 5/7 participants stated this to be generally the case, and one found it to be distinctive for his/her depressive episode. In the decrease group 8/15 participants stated a general deceleration, and seven found it to be specific to their depressive episode. Most participants reported either an increase or a decrease in velocity corresponding to the onset of the current depressive episode; however, two participants gave statements revealing both an increase and a decrease since exacerbation, with one stating the increase to occur in retrospect.

Three categories described changes in the passage of time non-related or not specifically related to velocity (categories 06, 08, 09; in total: *n* = 20 statements, app. 8.5% of all statements). Participants reported a normalization or improvement of the experience of passage of time while being engaged in social or pleasant activities (category 06, *n* = 6 statements, 24% of participants). The same number of participants reported an inability to feel, perceive or give meaning to the passage of time (category 08, *n* = 5 statements, 20% of participants). Relatedly, participants further stated the experience of the passage of time being inexorable, endless, circular or a standstill (category 09, *n* = 9 statements, 36% of participants). Additionally and in contrast to various statements mentioned before, several depressed participants reported the absence of situational influence on the passage of time (category 07, *n* = 9 statements, 36% of participants).

### Category group “present”

Concerning the Experience of the Present, several categories described the present as enduring (categories 12, 15, 17; in total *n* = 19 statements, 8% of all statements). Several participants stated that the present was experienced as extended (category 17, *n* = 10 statements, 40% of participants). Correspondingly participants gave account of the present being a current activity (category 15, *n* = 6, 24% of participants) or being contained within 1 day (category 12, *n* = 3 statements, 12% of participants). Concordantly, participants described the present taking place as a conscious„ now” or within a current moment (category 13, *n* = 7 statements, 28% of participants). Strikingly, a significant number of participants experienced the present as a point in time instead of an extended duration (category 16, *n* = 10 statements, 40% of participants). One category of statements concluded that the present could be influenced (category 19, *n* = 7 statements, 28% of participants), however the identical number of participants judged the present to be out of their own control (category 18, *n* = 7 statements, 24% of participants). Nine participants judged the present to be related to negative emotions (category 14, *n* = 9 statements, 36% of participants), and the present to be meaningless (category 20, *n* = 5 statements, 20% of participants).

### Category group “past”

Within the group concerning the Experience of the Past participants stated that the past had a negative effect on the present and/or was dominated by feelings of guilt (category 21, *n* = 15 statements, 60% of participants). Additionally, participants experienced the past as over and unchangeable (category 22, *n* = 10 statements, 40% of participants) and that the past had to be accepted (category 23, *n* = 3 statements, 12% of participants). Ten participants stated that the past had influenced and shaped their personality or that it could be learned from (category 24, *n* = 10 statements, 40% of participants).

### Category group “future”

Within the group Experience of the Future statements describing the future as being related to negative emotions such as fear, worry, and hopelessness were identified most frequently (category 25, *n* = 10 statements, 40% of participants). Surprisingly, the same number of participants referred to the future as being related to thoughts of hope (category 29, *n* = 10 statements, 40% of participants). Slightly fewer statements referred to a diminished, blocked future, meaningless, and no longer part of subjective experience (category 26, *n* = 8 statements, 32% of participants), or uncertain, non-projectable, and out of one's own control (category 27, *n* = 7 statements, 28% of participants).

## Discussion

The results obtained from content analysis confirmed the very few retrospective studies (Stanghellini et al., 2016, 2017), case reports and theoretical accounts of a disturbance of the experience of time in MDD. Whereas the overall nature of a fluent and variable passage of time seemed to be intact with acceleration in pleasant and deceleration in unpleasant situations (Vogel et al., in revision), depressed patients showed general changes in their experience of the passage of time. The most common was that of time having slowed down, most reliably found experiential disturbance in MDD within the literature (Thönes and Oberfeld, 2015).

Seemingly contradicting, we also found statements on an increase in the experienced velocity of the passage of time. Although we cannot rule out completely hypomanic or manic experiences, an involvement of corresponding symptoms seems to be highly unlikely, as pre-inclusion diagnostic procedures were conducted rigorously according to clinical guidelines, and all participants showed clear depressive symptoms in a clinical range as additionally assessed by BDI. We therefore speculate that this apparent increase may be due to one or more out of three conceivable associations. First, stands the possibility of a retrospective judgement of the duration of the past course of the depressive episode—a phenomenon found within the data and which would be describable by the so called *time paradox* (Wittmann, 2016, also see Straus, 1928). According to this phenomenon, uneventful durations (in this case the depressive episode) retrospectively are judged to have been shorter than real time or the contained time seemed to have gone by more quickly than real time.

Second, clinical improvements of participants after having started treatment may explain statements of an increase in the overall speed of the flow of time. The ostensible discrepancy in patient reports possibly and in part may have been influenced by the heterogeneous treatment regimen found in our patient group. Furthermore, it has recently been shown, that the experience of time passing quickly can be found in healthy individuals (Vogel et al., in revision). In other words, the increase in the velocity of the passage of time would not appear to be a distinct feature to MDD but in our case more closely reflected decreasing symptom severity.

Third, may stand a discrepancy between the description of *internal time/ego-time* and *external time/world-time* (Straus, 1928; Lehmann, 1967; Minkowski, 1971) or *intersubjective time* (Fuchs, 2013), with some subjects describing their own passage of time as slowed down, and fewer subjects describing the external world time as moving faster and passing by. Concordantly, our finding of both an increase and a decrease in time in MDD may effectively describe the same phenomenon from two different points of view (internal vs. external). As a speculation, this finding may describe two psychopathological subgroups of MDD, with one group comparing external time to internal time (e.g., “everyone is passing me by”), causing the velocity of the flow of time to be described as increased, or comparing internal time to external time (e.g., “I am slower than everyone else”), causing the velocity of the flow of time to be described as decreased. In a simpler line of reasoning the differing reports on the velocity of the flow of time, may psychopathologically delineate MDD subgroups with decreasing velocity being the well-known more prevalent phenomenon and an increase in velocity posing a newly described symptom, specific to a subordinate division of depressive disorder.

When focusing on the aspect of context-dependency of the passage of time we find a discrepancy between the number of statements stating that the passage of time will speed up when engaged in a pleasant activity or situation and that it will slow down when engaged in an unpleasant activity or situation. Although our method is not entirely suitable for quantitative evaluation, it relates to a somewhat similar finding, which has very recently been reported in the judgement of the velocity of the experience of time in an Experience Sampling Method (Dupuy, 2017). The authors were unable to find a significant reduction in the subjective speed of time in unpleasant situations in participants with MDD. We argue that it seems likely that an individual in a depressed state will not report any further deceleration in the velocity of the passage of time in an unpleasant situation due to the overarching unpleasantness of the depressed state and the related deceleration of the passage of time.

Regarding intersubjective and social aspects of time experience our results are heterogeneous. We speculate that an increase in the velocity of the passage of time in the presence of others, just as the reported normalization of its experience relative to situation, reflects that social interactions are usually experienced as pleasant and engaging, therefore related to an improvement in affect or mood, which in turn causes the passage of time to be experienced as moving faster (Vogel et al., in revision). It is important to note that our method of qualitative inquiry into inner experience is insufficient to provide proof of *implicit* interpersonal aspects of time experience and *temporal desynchronization*, which have previously been convincingly conceptualized as a possible underlying mechanism of disturbances in time experience in depressive disorders (Fuchs, 2001, 2005, 2013; Ratcliffe, 2012).

Concerning what has been referred to as the experience of the *structure of time* (i.e., past, present, and future) (Kupke, 2009; Vogel et al., in revision), we—similarly to the passage of time—find an overall conserved nature of experience of time structure, with the notion of an experience of time advancing from the past, through the present, into the future. As previously observed in healthy individuals (Vogel et al., in revision), and as opposed to persons suffering from schizophrenia (Vogeley and Kupke, 2006; Stanghellini et al., 2016), participants with MDD seem to retain this structured directedness of time from the past to the future. In this context, both depressed and healthy individuals experience their lives in the present, where it may be possible to influence the future through a present activity drawing from previously acquired knowledge and past experiences. However, several distinct features arise from our analysis. It can be concluded that depressed participants experience a diminished ability to influence the present and the future. It seems as if they feel detached from the otherwise intact structured directedness of time. Time seems unchangeable, and the present is reduced to circling (daily) repetition. It can further be shown that the present and the past are strongly experienced as negative and in the former case as meaningless. Our participants experienced the past as a source of guilt and as providing the reasons for one's present suffering. The future was experienced as *blocked*; to our participants it seemed out of reach and meaningless. Both the experience of the future being uncertain and of the future being experienced as frightening seemed much more pronounced than previously observable in healthy individuals (Vogel et al., in revision). Both experiences of uncertainty and fear carried with them additional feelings of hopelessness and inevitability. These findings are in good concordance with theoretical considerations on the psychopathology of time in depression. The above mentioned disturbance of *lived time* has also been referred to as *blocked future* (Straus, 1928; von Gebsattel, 1928; Fuchs, 2001; Wyllie, 2005; Stanghellini et al., 2017), *disturbance of (vital) becoming* (Straus, 1928; von Gebsattel, 1954; Minkowski, 1971; Fuchs, 2013), *disturbance of protention* (Binswanger, 1960), or *disorder/disturbance of conation* (Stanghellini et al., 2017). In simple terms, this terminology describes the depressed patient's inability to advance into the future through goal directed or planned action. This subsequently renders the future inaccessible, turning the past into the dominant temporal domain. Subsequently, past experiences are deemed responsible for the depressed present state. Statements within our data conveying an experience of a decreased or diminished ability to influence the present and the future, an experience of an intangible future further foregrounded by a slowed down, halted or endless passage of time, and the addition of feelings of guilt and negativity to an otherwise still influential past, highly correlate with the hypothesized disorder of *lived time*. Although it was not possible to draw conclusions as to the dynamic of the onset and interrelationship of depressive symptoms of time experience from our data, we conclude that the experience we captured by employing the presented method, empirically validates MDD as a disorder of *lived time*, including its correlating symptoms.

Our findings coincide with conclusions from a study by Stanghellini et al. (2017) on the Abnormal Time Experience (ATE) in MDD. The study found three changes in temporal experience, namely (1) a standstill of bodily functions (“vital retardation”), (2) a present and future dominated by the past, and (3) a slowing or blocking of the flow of time (“slackening of the flow of time”). The second category roughly corresponds to statements of guilt and the negative influence of the past. The third category corresponds to statements on the decrease in velocity of the passage of time, the standstill and endlessness of the passage of time. However, we were unable to detect statements referring to the first category of ATE. This is most likely due to the fact pointed out by Stanghellini et al. (2017) that this category refers to more implicit changes of time experience. Hence, it will most likely not be detectable with a questionnaire explicitly inquiring the experience of time.

Observations and analyses leave us with a coherent account of disturbed time experience in MDD. As clearly represented in Table 4, participants with MDD experience an inability or difficulty to influence or advance from a meaningless and unpleasant present. Their future seems blocked and the past overwhelmingly negative. These phenomena present themselves to the depressed individual before the unaffected background of a concept of time, meaning that time not only keeps its structured directedness, but for the individual suffering from MDD the basic notion of how time was experienced before the onset of depression is preserved during illness. This background may arise from implicit intersubjective aspects of temporality, and be the distinguishing origin of depressive suffering (Ratcliffe, 2012; Fuchs, 2013).

**Table 4:** Disturbance of *Lived Time* in depression.

**Symptom**	**Definition**	**Relating categories**
Slowed/halted flow of time.	The passage of time feels unpleasant; time seems to have slowed down or stopped; the patient has to struggle against time and its flow.	K07, K08, K09, K11, K20
Meaningless present.	The present holds no significance; it cannot be influenced sufficiently; it seems endless or circular.	K09, K14, K19, K20
Overwhelmingly negative past.	The past's influence is exceedingly negative; feelings and thoughts of guilt and sorrow dominate; former misdoings cannot be redeemed.	K21, K22, K23, K26
Blocked future.	The patient feels stuck in depressive suffering; change and advances are not possible; the future is meaningless and hopeless.	K09, K21, K25, K26

The syndrome of disturbed *lived time* in MDD emerging from our analysis suggests a variety of implications for further research with both phenomenological and cognitive neuro-scientific approaches. The relationship between the inseparable formats of time experience—passage of time and structure of time—remains largely uninvestigated in phenomenological, cognitive and in neuroscientific research. The syndrome of a disturbed *lived time* in MDD demonstrates convincingly the intricate relationship of the passage or flow of time and the structure of time. Neither the experience of the passage of time, nor the experience of the dimensions of time, i.e., past, present, and future, are clearly separable. They interpenetrate and influence each other in a dynamic, complex and “dialectic” way, creating what we refer to as structured directedness.

We speculate that changes in the experience and judgment of the subjective flow of time reflect an individual's present state of becoming, i.e., the congruence of the present activity or state and a desired state. Thus, an individual will experience an unpleasant deceleration in the flow of time during an unpleasant or non-engaging activity (Zakay, 2014), or an acceleration in the flow of time or pleasant fading out of the experience of time (e.g., Csikszentmihályi, 1990; Hancock and Weaver, 2005) during pleasant or engaging activities. The same may rule true for mental disorders and especially affective disorders. Inversely, it has been discussed that the experience of the velocity of the passage of time may be causative to a pleasant or unpleasant experience during a specific activity (Sackett et al., 2010); transcribed to MDD this would suggest that a decrease in the velocity of the passage of time is causative to depressed mood. In either case it seems plausible that the mechanism behind time experience serves as a specific indicator for the current state of what has been referred to as an individual's becoming (Minkowski, 1923, 1971; Straus, 1928; von Gebsattel, 1954; Fuchs, 2013), and that experiences of velocity changes are an integral component of the disturbance of *lived time* in MDD (Straus, 1928). Relatedly, the subjective speed of time passage has been put forward as reflective of overall status of health (Droit-Volet, 2013; Droit-Volet and Wearden, 2016).

It has recently been proposed that the repeated findings of a subjectively slowed down flow of time in MDD, when contrasted with the heterogeneous findings from time perception research in depressive disorders, in addition to other diverging results between subjective time experience and timing, may be due to two separate underlying mechanisms (Droit-Volet, 2013; Lamotte et al., 2014; Droit-Volet and Wearden, 2016). The mechanism for time perception being the heavily investigated *internal clock* (Church, 1984; Droit-Volet, 2013; Shi et al., 2013; Allman et al., 2014), and the mechanism for time experience still remaining unclear, although introspection has been suggested as a possible underlying process (Fayolle et al., 2013; Lamotte et al., 2014). These disturbances of subjective time experience have been associated with impaired decision-making (Wittmann and Paulus, 2008; Woods et al., 2014; Owen et al., 2015). Additionally, a slowed down subjective flow of time has been conceptualized to be linked to psychomotor retardation in MDD (Kitamura and Kumar, 1982; Blewet, 1992; Bschor et al., 2004; Gil and Droit-Volet, 2009; Stanghellini et al., 2017), although diverging findings exist (Bech, 1975).

As changes in the brain's resting state (Kaiser et al., 2015; Drysdale et al., 2017) have been implicated as possible underlying neuropathophysiology in MDD, it has been speculated, that phenomenological (spatio-)temporal abnormalities and (spatio-)temporal abnormalities observed through neuroimaging in psychiatric conditions including MDD may be corresponding phenomena (Northoff, 2016a,b; Northoff and Stanghellini, 2016). We argue that in the case of MDD, the psychopathological specifiers lie in the disturbance of *lived time*.

In a differing context resting state data has provided results for further differentiation of subtypes in MDD (Drysdale et al., 2017; Price et al., 2017) and in line with current opinion we suggest that psychopathology may offer a complementary approach to neuroscientific proceedings in identifying secondary types or specifiers of MDD and vice versa (Sullivan et al., 2002; Fountoulakis et al., 2004; Stanghellini and Rossi, 2014; Owen et al., 2015; Stanghellini and Aragona, 2016). In accordance with our findings, we suggest a possible subgroup distinction may lie in reports of experiences of an increase and a decrease in the velocity of the flow of time. As specified above, accounts of a slow flow of time may be specific to more severe depressed states and those about fast flow of time may either reflect recovery from depression, hence possibly posing a specific symptom to less severe depressive episodes, or it may in fact be a distinctive feature to a previously unidentified clinical subtype of MDD. Unfortunately, due to the limitations of qualitative analysis - specifically vulnerability to false negatives, possible oversimplification, and the only partially quantitative assessment of categories—, it is not feasible to search for the assumed correlations between the occurring symptoms of disturbances in subjective time and e.g. overall depressive symptom severity on the basis of our data. Although we consider the sample size saturated, fully adequate and sufficient for our qualitative assessment (Sandelowski, 1995; Pope et al., 2000; Mason, 2010; Glaser and Strauss, 2017), it is too small to search for discrete subtypes within the identified syndrome, as the qualitative method is not intended for this type of statistical investigation (Brown and Lloyd, 2001). Further integrative research approaches will be necessary to properly examine the possibility of distinct depression subtypes within the corresponding disorder of subjective time experience.

## Limitations and conclusion

Using a time questionnaire (TQ) specifically designed for the investigation of time experience, we acquired qualitative material from 25 participants suffering from MDD. Although patients were recruited after a rigorous diagnostic process, we did not administer psychometric procedures after inclusion and the treatment regimen was relatively heterogenic. This possibly may have interfered with patients' self-reports. These limitations of our study are accompanied by general methodological limitations of content analysis, primarily vulnerability to false negatives, possible oversimplification, and the only partially quantitative assessment of categories. Despite these restrictions, we were able to identify a specific disturbance of *lived time* and corresponding clinical symptoms in MDD. These findings hold several implications. As disturbances in time experience in MDD have been repeatedly described in previous literature, but as of yet have not been included as diagnostic criteria, the detailed description of an underlying syndrome provided herein, might facilitate additional diagnostic accuracy. Moreover, our findings suggest a probable specification of MDD subgroups based on the disturbance of *lived time*, be it between degrees of severity or genuinely distinct depressive syndromes. Further research will be needed to investigate the implications posed by a syndrome of disturbed time experience in MDD. We suggest that integrative research designs combining both psychopathological and neuroscientific methods will provide the most fruitful approach to the further investigation of time experience.

## Ethics statement

This study was carried out in accordance with the recommendations of the Ethics Commission of the Faculty of Medicine of Cologne University with written informed consent from all subjects. All subjects gave written informed consent in accordance with the Declaration of Helsinki. The protocol was approved by the Ethics Commission of the Faculty of Medicine of Cologne University.

## Author contributions

DV, CK, and KV: Conceptualization; DV: Data Curation; DV: Investigation; DV: Qualitative analysis; DV, KK, and TS: Coding and Intercoding; DV, TS, and KK: Statistical Analysis; KV: Supervision; CK and KV: Resources; DV: Writing—original draft; DV, TS, CK, and KV: Writing—review and editing.

### Conflict of interest statement

The authors declare that the research was conducted in the absence of any commercial or financial relationships that could be construed as a potential conflict of interest.
